# The PALLI-AI Framework: An Evidence-Informed Checklist for Designing and Reporting Trustworthy Artificial Intelligence Tools in Palliative and End-of-Life Care

**DOI:** 10.3390/healthcare14182998

**Published:** 2026-09-14

**Authors:** Juan Mora-Delgado, Víctor Rivas Jiménez, Cristina Lojo-Cruz

**Affiliations:** 1 Department of Infectious Diseases and Clinical Microbiology, Hospital Universitario de Jerez de la Frontera, 11407 Jerez de la Frontera, Spain; 2 Instituto de Investigación e Innovación Biomédica de Cádiz (INiBICA), 11009 Cádiz, Spain; 3 Department of Internal Medicine and Palliative Care, Hospital Universitario de Jerez de la Frontera, 11407 Jerez de la Frontera, Spain; 4 Spanish Society of Palliative Care (SECPAL), Paseo de La Habana 9–11, 28036 Madrid, Spain

**Keywords:** palliative care, end-of-life care, serious illness, early palliative care, illness trajectory, total pain, artificial intelligence, machine learning, large language models, checklist, reporting guideline, trustworthy AI

## Abstract

**Highlights:**

**What are the main findings?**

- We propose PALLI-AI, a 22-item checklist across seven domains that works as both a reporting standard for researchers and a design guide for developers, with every item drawn from a tension specific to serious illness rather than transplanted from generic guidance.
- Each item is framed with a dual lens: what to report in a publication and what to build into the tool.

**What are the implications of the main findings?**

- PALLI-AI names failure modes unique to end-of-life care, among them the self-fulfilling prognosis, the algorithmic surrogate and abandonment by automation, and it maps where it adds to existing AI reporting guidelines rather than repeating them.
- The framework offers a shared vocabulary for clinicians, methodologists, developers and regulators to appraise and improve trustworthiness before a tool reaches a patient living with serious illness.

**Abstract:**

**Background/Objectives:** Artificial intelligence (AI) is being applied across palliative and end-of-life care, including prognostication, symptom detection, clinical documentation, communication support, decision support and care coordination. General AI reporting guidelines exist, but none captures the value-sensitive requirements specific to palliative care, which is integrated from the diagnosis of a serious illness and spans the whole trajectory, alongside disease-directed treatment, to the end of life and bereavement. We aimed to develop an evidence-informed checklist to guide the design and the transparent reporting of AI tools in this setting. **Methods:** We conducted a narrative review of the literature on AI in palliative care, prognostic communication, surrogate decision-making, digital-health co-design, algorithmic fairness, explainability, AI governance and existing reporting guidelines (TRIPOD+AI, CONSORT-AI, SPIRIT-AI, DECIDE-AI). During revision, the search was formalised and re-executed in PubMed (25 August 2026), each item was graded for certainty of evidence, and the checklist was piloted on four published studies. Rather than reusing generic requirements, we derived each item from a tension specific to serious illness, named the failure modes those tensions produce, and mapped where the framework diverges from generic guidance. **Results:** PALLI-AI comprises seven domains—Purpose set by goals of care; Alignment with total suffering and dignity; Lived experience, family and bereavement; Learning data and the equity of access; Interpretability and prognostic communication; Accountability in serious illness; and Implementation and palliative outcomes—operationalised as 22 items, each with a researcher and a developer formulation. We name six failure modes specific to this setting, including the self-fulfilling prognosis, the algorithmic surrogate and abandonment by automation, and we set out, domain by domain, what the framework adds beyond existing guidelines. In the pilot, all items were ratable; safeguards against the self-fulfilling prognosis, decedent data governance and answerability were unreported across studies. **Conclusions:** PALLI-AI is a pragmatic, non-prescriptive checklist intended to raise the trustworthiness of AI tools for people approaching the end of life. It is a starting point for formal consensus development and empirical validation, not a finished standard.

## 1. Introduction

Palliative care is not confined to the final days of life. The concept of total pain, introduced by Cicely Saunders, framed the suffering of serious illness as physical, psychological, social and spiritual at once, and the modern consensus definition describes palliative care as the active, holistic care of people with serious health-related suffering, across all ages and stages, and not only those near death [1]. It is applicable early in the illness course and alongside disease-directed treatment: in a randomised trial, palliative care integrated from the diagnosis of metastatic lung cancer improved quality of life and mood, reduced aggressive treatment at the end of life, and was associated with longer survival [2]. Need also unfolds along recognisable illness trajectories, from the late, steep decline of cancer to the intermittent crises of organ failure and the slow dwindling of frailty and dementia, each with its own tempo and its own timing of palliative input [3]. Any tool for this field must therefore serve the whole trajectory, of which the last phase of life is only one part.

Palliative care improves quality of life for people with life-threatening illness, yet access remains far below need. The Lancet Commission on Global Access to Palliative Care and Pain Relief estimated that tens of millions of people die each year with serious health-related suffering that could be relieved, most of them in low- and middle-income countries [4]. The World Health Organization estimates that, of the roughly 56.8 million people who need palliative care each year, only about 14% receive it [5]. Demand is rising: projections of serious health-related suffering among people dying from cancer alone forecast an increase from 7.8 million affected in 2016 to 16.3 million by 2060, with the steepest growth (a 407% rise) in low-income countries [6].

Two structural problems limit timely palliative care. Clinicians tend to overestimate survival, and specialist staff are scarce, so patients are identified late or not at all [7]. Machine-learning models trained on electronic health record data can flag patients likely to benefit from palliative input earlier than referral-based pathways [7,8]. In a cluster-randomised trial, an AI decision-support tool increased the rate of palliative care consultation (incidence rate ratio 1.44, 95% CI 1.11–1.92) and was associated with fewer 60- and 90-day readmissions [9]. Beyond prognostication, applications now span symptom detection, documentation, patient-facing education, communication support and care coordination [10,11,12,13], and large language models have entered clinical practice and education in the field, including nursing [14,15].

This growth has outpaced the guidance needed to design and evaluate such tools responsibly. General AI reporting guidelines cover prediction models (TRIPOD+AI), interventional trials (CONSORT-AI, SPIRIT-AI) and the early live evaluation of decision-support systems (DECIDE-AI) [16,17,18,19,20]. Broader governance frameworks set out principles for trustworthy and ethical AI in health [21,22]. None of these was written for the particular demands of caring for people with a limited prognosis, where the aim is relief of suffering rather than cure, where errors carry distinct harms, and where the human relationship is itself part of the treatment. A symptom-tracking app, a mortality predictor and a goals-of-care chatbot each raise questions that a generic checklist does not ask.

We propose PALLI-AI, an evidence-informed checklist for the design and reporting of AI tools in palliative and end-of-life care. It is built for two audiences at once: researchers, who need a reporting standard when they publish, and developers, who need the same requirements as design decisions before and during build. Its aim is not to repeat the generic guidelines but to add what they leave out. Each item is derived from a tension specific to serious illness, from a target set by goals of care rather than survival, to the communication of a prognosis, to the ethics of inferring the wishes of a person who may have lost capacity. This narrative review sets out the rationale, the seven domains and 22 items, names the failure modes the framework is designed to catch, maps where it diverges from existing guidelines, and states its limitations and the work needed to validate it.

## 2. Materials and Methods

### 2.1. Design, Search Strategy and Source Selection

This is a narrative review supported by a structured, reproducible search. During revision, we formalised the strategy and re-executed it in PubMed (National Library of Medicine, Bethesda, MD, USA; https://pubmed.ncbi.nlm.nih.gov) on 25 August 2026, from database inception, with no language restriction; the exact query strings, their PubMed translations and the record count of every block are given in Table S1. An initial exploratory search, run to July 2026, informed the original submission; the formalised strategy reported here was re-executed in full on 25 August 2026, and every count in this section refers to that definitive search. The core search combined a palliative and end-of-life block with an artificial-intelligence block and returned 872 records, of which 829 were published from 2015 onwards. Five targeted searches covered prognostic communication (19 records), surrogate decision-making and preference prediction (1796), algorithmic bias and fairness in health care (2799), explainable artificial intelligence in medicine (8368) and AI reporting guidelines (476). Reference lists of key reviews were hand-searched for additional sources. The literature review and the drafting of the search strategies were supported by the paperORnot platform (https://paperornot.com; accessed on 25 August 2026), an AI-assisted clinical-research tool; the final query strings, eligibility decisions and included sources were reviewed and verified by the authors.

Records were screened on title and abstract against explicit criteria. We included primary studies of AI applied to palliative or end-of-life care in any setting; systematic, scoping and rapid reviews of such applications; published AI reporting guidelines and guidance for their development; institutional governance documents from the World Health Organization and the European Union; and conceptual or ethical analyses bearing directly on prognostic communication, surrogate decision-making, algorithmic fairness or explainability in serious illness. We excluded conference abstracts without a full paper, studies of AI in oncology or intensive care without a palliative focus, and commentary that added no requirement not already captured by a stronger source. Within eligible records, selection was purposive, as befits a narrative review: sources were chosen for their capacity to define or illustrate a palliative-specific requirement, not to enumerate every study. Screening and selection were performed by one author (J.M.-D.) and independently checked by the other two; disagreements were resolved by discussion until consensus. The evidence base comprises 43 peer-reviewed publications and 4 institutional documents (47 references in total).

### 2.2. Framework Development

**Figure 1:**
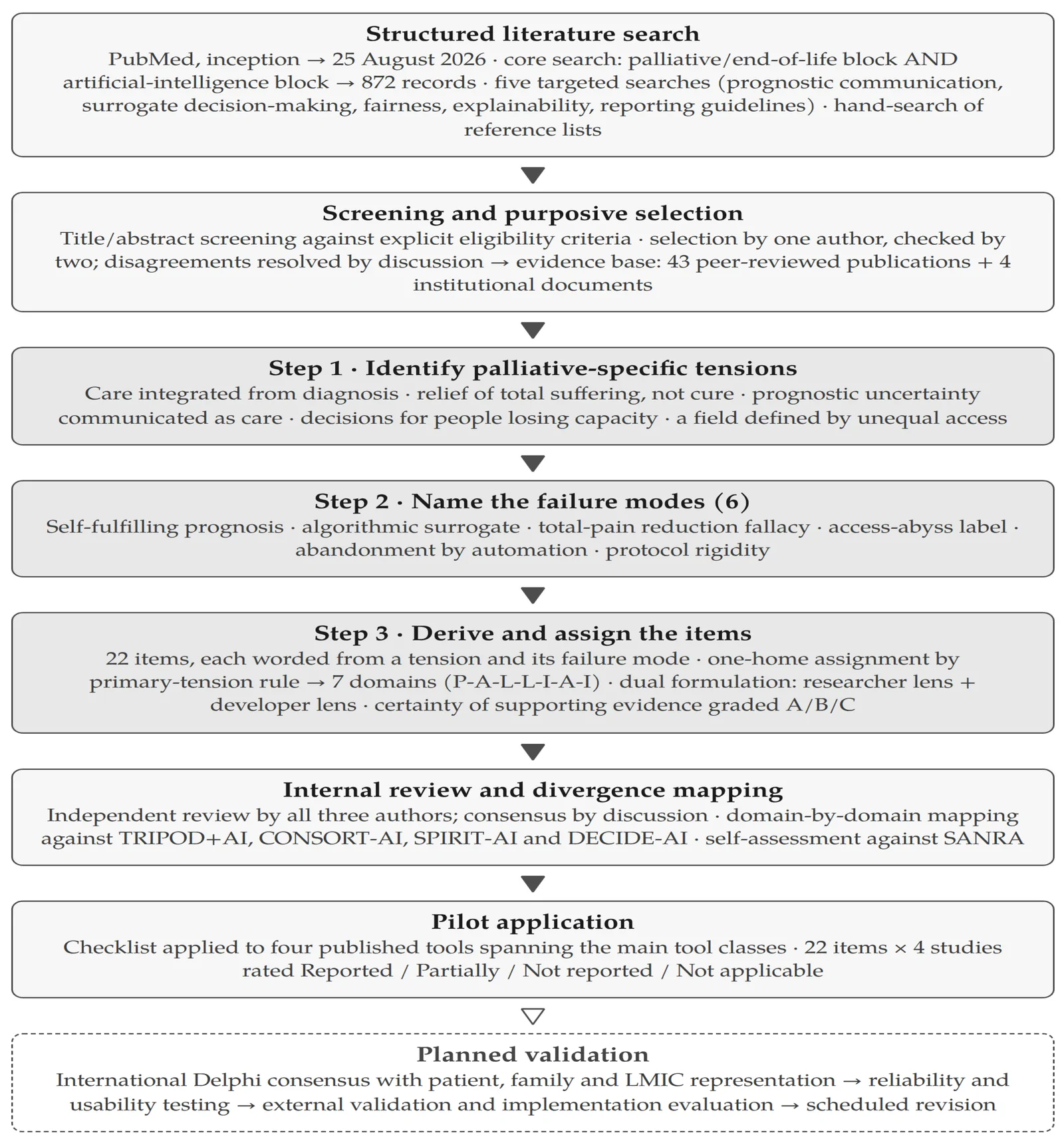
Development of the PALLI-AI framework: from the structured search and purposive selection, through the identification of palliative-specific tensions and failure modes, to the derivation and assignment of the 22 items, divergence mapping against generic guidelines and the pilot application. The dashed box shows the planned validation stages (Section 4.6). Solid arrows indicate the sequence of completed stages; grey shading marks the three derivation steps; the dashed border denotes planned stages. Full search strings and record counts are given in Table S1 and item-level pilot ratings in Table S3.

The framework was developed in three steps, summarised in Figure 1, and informed, where applicable to a pre-consensus proposal, by the EQUATOR guidance for developers of health research reporting guidelines [23]. First, we identified the tensions that make palliative care distinctive: care that begins at the diagnosis of a serious illness and is integrated with disease-directed treatment rather than reserved for the final days; a goal of relieving total suffering rather than achieving cure; prognostic uncertainty communicated to patients and families as part of care; decisions taken for people who may lose capacity; and a field defined by unequal access. Second, we named the recurrent failure modes these tensions produce, such as the self-fulfilling prognosis and the algorithmic surrogate, and used them to word the items. Third, we grouped the items into seven domains whose initials form the mnemonic PALLI-AI, expressed each as a reporting formulation for researchers and a design formulation for developers, and mapped, domain by domain, where the framework adds to generic guidance (Section 3.9).

Candidate items were drafted by one author (J.M.-D.) from the sources above. The other two authors (V.R.J., C.L.-C.) reviewed the candidate set independently; wording, merging and grouping were revised in response, disagreements were resolved by discussion until consensus, and all three authors approved the final set of 22 items. No formal voting procedure was used, and no external stakeholders, patients, caregivers, developers or regulators among them, took part at this stage; both limitations are examined in Section 4.5. Each item was assigned to exactly one domain by a primary-tension rule: it belongs to the domain named for the tension it exists to resolve, even where it touches others. Cross-cutting themes therefore recur by design rather than by oversight: patient preferences appear in Alignment as a value the tool must encode, in Interpretability as content it must render faithfully, and in Accountability as a limit on inference; equity appears in Learning data as a property of the training corpus and in Implementation as a property of scale-up; human oversight appears explicitly in Accountability and implicitly in non-abandonment, as presence at the bedside. The one-home rule keeps the checklist free of duplicate items, while the deliberate recurrence keeps each domain self-contained for a future rater. The reporting quality of this manuscript was self-assessed against the SANRA criteria for narrative reviews [24]. PALLI-AI has not undergone formal Delphi consensus; it is a structured proposal offered for appraisal and refinement.

### 2.3. Evidence Classification

For each checklist item we recorded the supporting sources, the palliative-specific tension it answers, the failure mode it guards against and a certainty grade for its evidence, at three levels: level A, direct comparative evidence, from randomised or controlled studies bearing on the requirement; level B, direct non-comparative evidence, from observational, qualitative, feasibility or review studies in palliative populations; and level C, conceptual evidence, from normative analyses, governance documents or findings in adjacent fields applied to palliative care by argument. The full evidence-to-item matrix is provided in Table S2. Of the 22 items, 3 are supported at level A, 14 at level B and 5 at level C, a distribution that is itself a finding: the requirements of trustworthy palliative AI rest mostly on observational and conceptual ground, and comparative evidence exists only where trials have linked prediction to referral and early integration to outcomes [2,9].

### 2.4. Pilot Application

To test whether the checklist can be applied to published work, we piloted it against four studies already discussed in this review, chosen to span the main tool classes: a deep-learning prognostic model, a cluster-randomised trial of a decision-support tool, a large-language-model documentation tool and a co-designed symptom-monitoring application. Each of the 22 items was rated against the publication as reported, partially reported, not reported or not applicable to the tool type, judging completeness of reporting rather than quality of the work. Ratings were drafted against the published reports and reviewed by all three authors, with disagreements resolved by discussion; we did not compute formal inter-rater statistics, which belong to the reliability study planned in Section 4.6. Results appear in Section 3.10 and item-level ratings with justifications in Table S3.

## 3. The PALLI-AI Framework

### 3.1. Rationale and Structure

**Figure 2:**
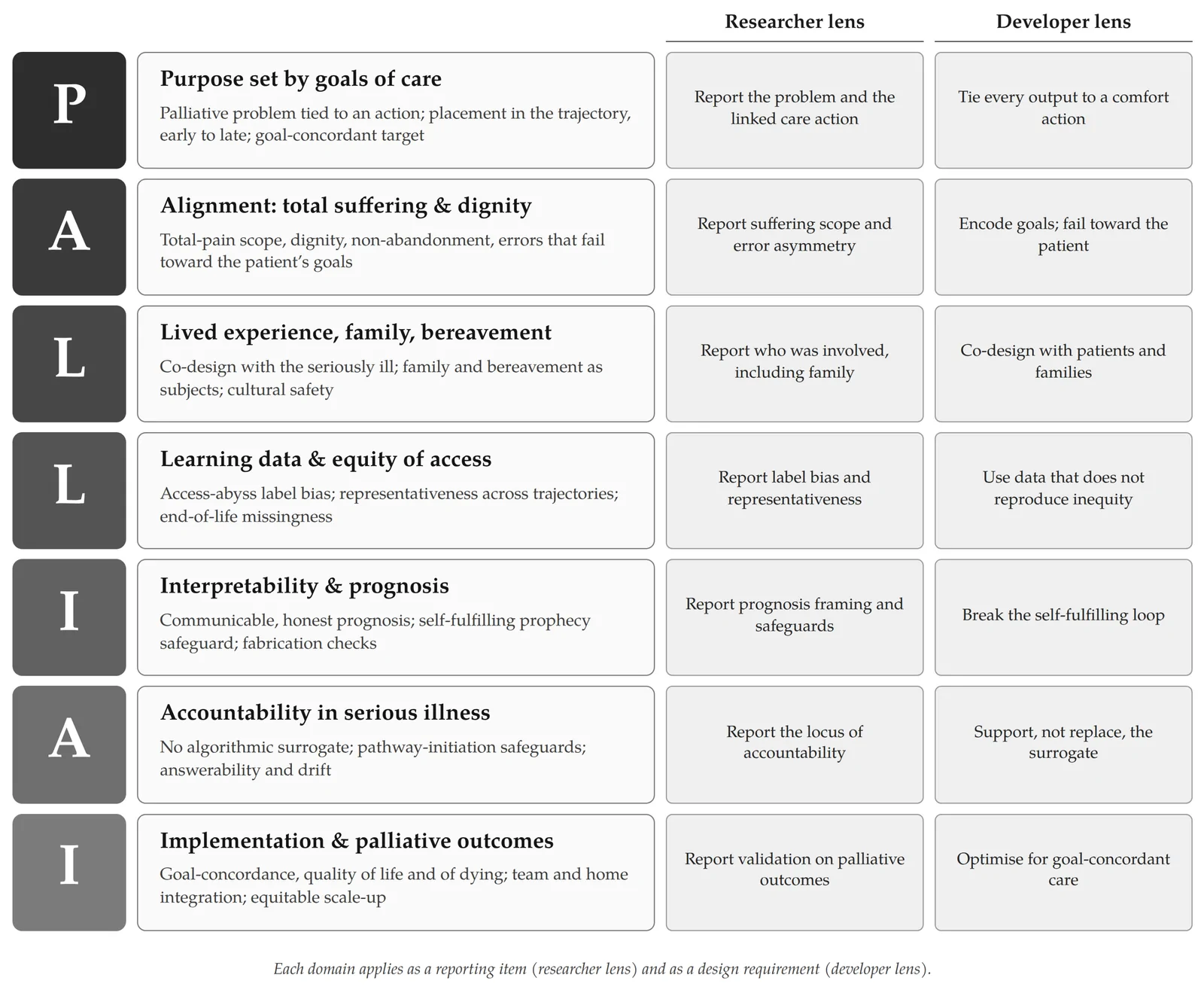
The PALLI-AI framework. Seven domains, each defined by a tension specific to palliative and end-of-life care. Their initials form the mnemonic, and each domain applies as a reporting item for researchers and as a design requirement for developers.

PALLI-AI is organised as seven domains that follow the arc of a tool from purpose to evidence, but each domain is defined by a palliative tension rather than by a generic development stage (Figure 2). Items were assigned to domains by the primary-tension rule described in Section 2.2, so cross-cutting themes such as preferences, equity and human oversight recur deliberately across domains rather than being confined to one. The domains are Purpose set by goals of care; Alignment with total suffering and dignity; Lived experience, family and bereavement; Learning data and the equity of access; Interpretability and prognostic communication; Accountability in serious illness; and Implementation and palliative outcomes. Twenty-two items are distributed across them (Table 1), and six named failure modes (Section 3.9) explain why the items are worded as they are. The framework is method-agnostic: it applies to a logistic-regression risk score, a deep-learning mortality model, a natural-language-processing pipeline and a large language model alike, because its questions concern purpose, values, data, communication, accountability and evidence rather than a particular algorithm.

### 3.2. Domain P—Purpose Set by Goals of Care

A trustworthy tool in this field starts from a goal of care, not from a prediction target. Three questions define its purpose. First, what palliative problem does it address, and what comfort or goal-concordant action does its output trigger? A prognostic score that is not tied to an action, a referral, an advance-care-planning conversation, or a symptom intervention is not a legitimate purpose in a setting where prediction for its own sake changes nothing for the patient. Second, where in the illness trajectory does it act? Palliative care is integrated from the diagnosis of a serious illness alongside disease-directed treatment, and it follows different trajectories, from cancer to organ failure to frailty, each with its own tempo [2,3]; a tool built only for the last days of life addresses a small part of that span, so its intended point on the trajectory, whether early identification, the management of an advanced but stable illness, or the final phase, should be stated rather than assumed to be the end. Third, is the target justified? Scoping reviews of AI in home-based serious illness and in nursing homes find that most tools remain at the feasibility stage and specify the clinical problem loosely [11,12]. Naming the target as a palliative outcome and stating explicitly that survival, cost or resource use are not the sole proxy for need, guards against the most common category error in this literature.

### 3.3. Domain A—Alignment with Total Suffering and Dignity

Palliative care attends to total suffering, the concept introduced by Cicely Saunders in which physical, psychological, social and spiritual pain are experienced as one, and its goals are set by the person [1,4]. A tool should state which of these dimensions it addresses and declare any single-dimension proxy, because a physical symptom score cannot stand in for existential or spiritual distress; the active ingredients of palliative care include existential and spiritual support, not symptom control alone [25]. Alignment also concerns the direction and the asymmetry of the tool’s errors. In most of medicine, a false negative that delays treatment is the graver error; here the calculus differs, because a false positive that drives reflexive escalation, or a false negative that permits premature withdrawal, can each add suffering or hasten death. A tool should state whether it pushes toward comfort and de-escalation or toward intervention, and justify its thresholds in those terms. Finally, the therapeutic relationship is itself part of the treatment. Commentary in the field warns repeatedly that AI must complement, not replace, human presence [14,26]; we therefore treat non-abandonment as a design requirement, so that automating a task frees clinician time for the person and family rather than removing the clinician from them.

### 3.4. Domain L—Lived Experience, Family and Bereavement

The people who should shape the tool are the seriously ill, their families and the clinicians who care for them. Co-design in palliative care is feasible and improves relevance: a remote-monitoring system co-designed with patients and health workers, and a symptom app co-created through community-partnered participatory research, were both judged usable by their intended users, and sustained involvement is possible even among older adults often assumed to be disengaged [27,28,29]. The setting imposes its own methods, because participants may be frail, may lose capacity and may die during the study, so involvement has to be designed for people who can give only a little. Two features distinguish this domain from generic user-centred design. The family is a unit of care and a data subject in its own right, before the death and into bereavement, so caregiver outcomes and the governance of family data belong in scope. And the disclosure of prognosis, spiritual care and the practices around dying vary across cultures, so a tool built on one norm can harm under another; cultural and disclosure safety should be configurable rather than assumed.

### 3.5. Domain L—Learning Data and the Equity of Access

Data determine what a tool can do fairly, and in this field the data carry a specific distortion. Because most people who need palliative care never receive it [4,5], a label such as ‘received specialist palliative care’ or ‘enrolled in hospice’ records who reached the service, not who needed it, so a model trained on that label learns to reproduce the access abyss rather than to correct it. The lesson of the population-health algorithm dissected by Obermeyer and colleagues applies directly: an outcome chosen for convenience encoded a large racial bias, and correcting the target raised the share of Black patients flagged for extra help from 17.7% to 46.5% [30]. Four sources of inequity are worth distinguishing, because each calls for a different mitigation. Target or label bias arises when the outcome variable itself encodes access rather than need, as above. Selection bias arises when the training cohort under-represents those least likely to reach services, the old, the frail and the non-cancer trajectories among them [12,31]. Historical inequity arises when discriminatory patterns of past care are learned from otherwise accurate records, the mechanism of the population-health example [30]. Deployment inequity arises after release, when performance or adoption differs across settings and groups, so that a tool accurate on average still widens the gap it was meant to close. An analysis under item 11 should state which of these four it has examined and how. Fairness in medical algorithms is not a single property but spans the data, the model and the deployment context, and demands deliberate mitigation rather than assumed neutrality [32,33]. The palliative population is also old, frail, multimorbid and often non-cancer, and is under-represented in many datasets; age-related bias is documented and its mitigations remain few [31], and nursing-home and non-cancer trajectories are thinly covered [12]. A further problem is structural: near the end of life, the sickest patients stop generating data, so records are missing-not-at-random in ways that bias any model that ignores the mechanism. This domain therefore asks for analysis of label bias, representativeness across trajectories and settings, explicit handling of end-of-life missingness, and lawful governance of the data of people with advanced illness and of bereaved families, including after death [22,34].

### 3.6. Domain I—Interpretability and Prognostic Communication

In palliative care, the output is often a prognosis, and a prognosis is not merely displayed; it is communicated to a patient or a family as part of care. Interpretability here has three parts. The first is honest communication of uncertainty: an estimate presented without calibration or a range invites false certainty at the moment when candour matters most, which is why interpretable prognostic models pair predictions with explanations [7]; drawing on explainable-AI methods that have matured across biomedical research and acute-care medicine [35,36], the tool should render its output in a form a person can receive, and should say who communicates it. Reporting should therefore include the calibration of the model in the deployment population and across illness trajectories, not only its discrimination; the form in which uncertainty is conveyed, favouring ranges or best-case, worst-case and most-likely framings over bare point estimates; the explanation method used and evidence on the fidelity of its explanations, since a plausible explanation that misrepresents the model misleads precisely when trust matters most [35,36]; and the layering of outputs, because what a clinician needs, what a family can receive and who delivers each are distinct design decisions. The second is a hazard largely absent from generic guidance, the self-fulfilling prophecy. A pessimistic prediction can cause the outcome it forecasts by prompting withdrawal of treatment, and machine-learning models that predict withdrawal of life-sustaining therapy risk hard-wiring that loop, since institutional withdrawal culture, not the patient’s physiology, can drive the decision [37]. A tool must be designed so that its prediction does not silently trigger the withdrawal that confirms it. The third applies to generative tools: they must be grounded in the record, checked for fabrication, and faithful to the person’s documented wishes. Large language models can identify advance-care-planning content with high sensitivity but also fabricate, which is why a hallucination index and a low but non-zero fabrication rate were reported for one such system [38], and summaries of palliative consultations vary in fidelity across models [39].

### 3.7. Domain A—Accountability in Serious Illness

Someone must remain answerable when a tool shapes decisions in serious illness, and two accountability problems are specific to this field, both of which are sharpest as the illness advances. The first is the algorithmic surrogate. Tools that infer what a patient would want, the patient preference predictor and its personalised, language-model variant, are proposed to support substitute judgement for people who have lost capacity [40], but critics argue that satisfying an inferred preference is not the same as respecting the person’s autonomy, and a scoping review finds the arguments against currently outweigh those in favour [41,42]. A tool should support, not supplant, the surrogate and the person’s documented wishes, and should state how capacity is handled. The second is protocol rigidity. The Liverpool Care Pathway was a well-intentioned framework for care of the dying that, applied as a mandatory protocol, contributed to harm and was withdrawn after an independent review recommended replacing it with individualised priorities of care [43]. An AI tool that starts or withdraws an end-of-life pathway must require explicit clinician authorisation, keep a reversible and audited trail, and never harden into a box-ticking mandate. Around these sit the general duties that this population makes acute: a named accountable owner, the correct regulatory class under the European Union’s AI Act, and post-deployment monitoring for drift and for any signal of hastened death, because casemix and pathways shift over time [21,22,44]; embedding responsible-AI practices from the outset helps anticipate and resolve the ethical issues these tools raise [45].

### 3.8. Domain I—Implementation and Palliative Outcomes

Trustworthiness is demonstrated in the outcomes that matter to patients and families, not in discrimination metrics. The evidence base is still thin: a rapid review found only three machine-learning studies using routine data for end-of-life care, all limited to mortality prediction [8], and reviews of AI in supportive and palliative care and in cancer nursing conclude that prospective validation is scarce [10,46]. The cluster-randomised trial of an AI referral tool is the template, because it measured consultation rates and readmissions rather than accuracy [9]. The endpoints that count are goal-concordant care, recognised as the most important outcome in serious-illness research and now measurable from clinical notes in ways AI could scale [47], together with quality of life and symptom burden across the illness course, as improved by early integration [2], concordance between preferred and actual place of death, quality of dying and death, and avoidance of aggressive treatment in the last weeks. Implementation is also a question of reach and of team. Palliative care is delivered by an interdisciplinary team across hospital, home and hospice, so a tool must fit that whole workflow and be sustainable in under-resourced services [13,25]; and because most unmet need lies where resources are least [4,6], scale-up should be judged by whether it narrows or widens the access gap [34]. Open, registered, reproducible reporting, with code or model available where feasible and a statement of how AI was used, closes the loop.

### 3.9. Failure Modes Specific to Palliative AI

The domains are easier to apply once the recurrent ways these tools fail are named. We identify six failure modes that a generic checklist does not anticipate. The *self-fulfilling prognosis* occurs when a pessimistic prediction causes the death it forecasts by prompting withdrawal of treatment that is then read as confirmation; models that predict withdrawal of life-sustaining therapy are especially exposed because institutional culture rather than physiology can drive the decision [37]. The *algorithmic surrogate* occurs when a tool’s inference of a patient’s wishes displaces the person’s documented preferences or the human surrogate, turning a support into a substitute for autonomy [40,41]. The *total-pain reduction fallacy* occurs when a tool collapses psychological, social and spiritual suffering into a physical proxy it can measure, then reports on suffering as though the proxy were the whole [4,25].

The *access-abyss label* occurs when a model is trained on who currently receives palliative care and so encodes existing under-referral of the under-served as if it were ground truth [4,30]. *Abandonment by automation* occurs when efficiency gains reduce the human presence that is itself part of the treatment, so that a tool which saves time at the bedside removes the clinician from it [14,26]. *Protocol rigidity* occurs when a tool hardens into a mandatory pathway that overrides individual judgement, the error that led to the withdrawal of the Liverpool Care Pathway [43]. Each maps to one or more domains, and each is the reason the corresponding items are worded as they are. Table 2 sets out, domain by domain, what generic AI guidelines already cover and what PALLI-AI adds, so the boundary between the two is explicit. Table 3 operationalises the six failure modes, giving each a working definition, observable indicators, an example and a counterexample from the literature reviewed here, and a decision rule for whether the mode applies to a given tool.

### 3.10. Applying the Framework: Illustrations and a Pilot on Four Published Studies

Two brief illustrations show how the checklist discriminates between tools. A deep-learning inpatient mortality model used to trigger palliative referral can satisfy Purpose, Learning data and Implementation if it reports its population, calibration and a prospective effect on referrals and readmissions [7,9]. The framework then surfaces what such a model usually neglects: whether mortality is an acceptable proxy for palliative need (Alignment), how the risk is communicated and whether it could become a self-fulfilling prophecy (Interpretability), and who acts on the alert and answers for a false positive (Accountability). A goals-of-care large-language-model assistant inverts the profile. It may excel at Lived experience if co-designed with patients and families, but its principal risks are the algorithmic surrogate and fabrication (Accountability and Interpretability), its target must be the person’s goals rather than a default (Alignment), and its evidence requirement is a study of communication quality and goal-concordant care, not a readability score [38,39,47]. In each case the neglected domains are the ones that matter most for the person and their family.

**Figure 3:**
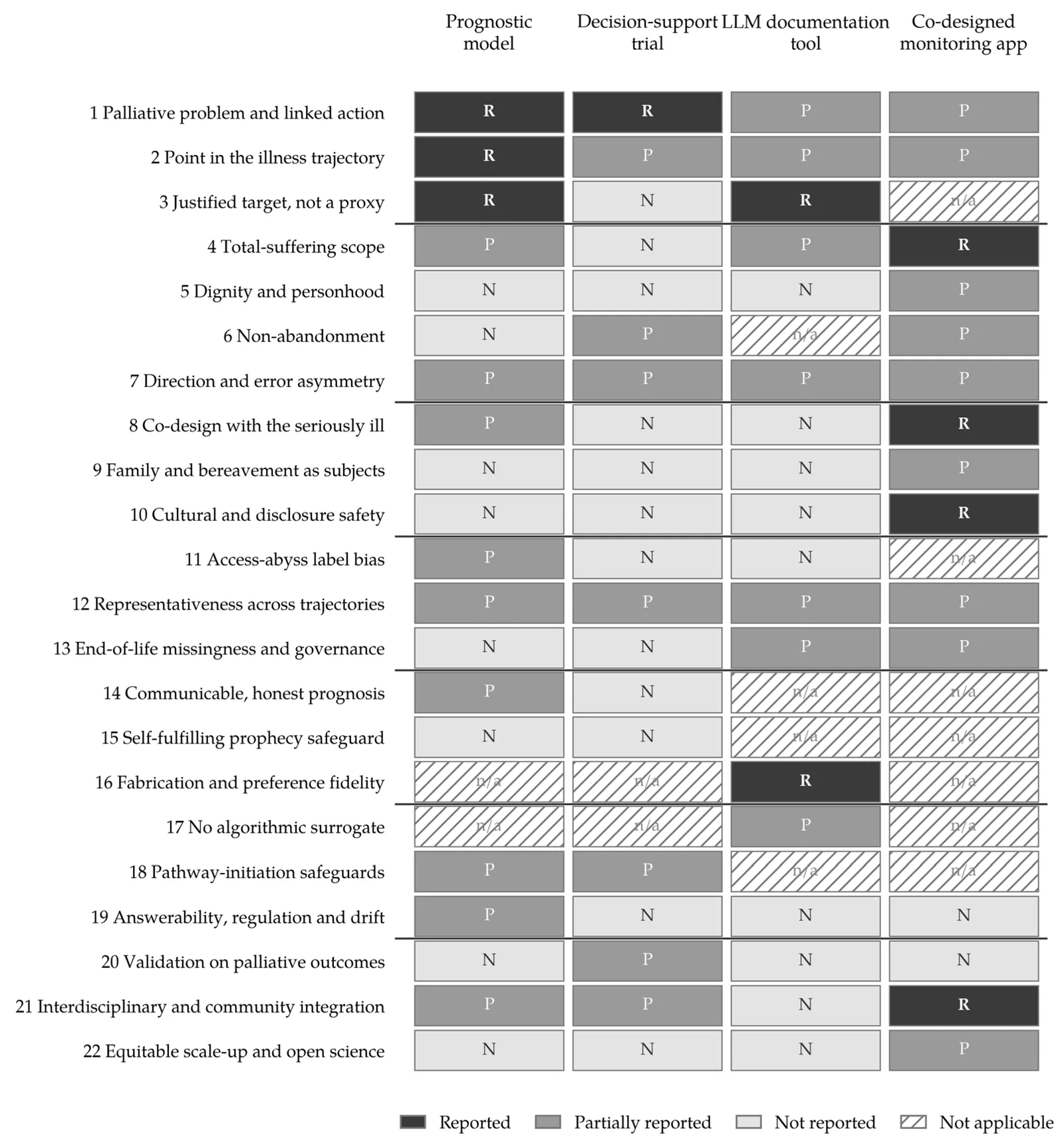
Pilot application of the 22 PALLI-AI items to four published AI tools in palliative care. Cells show whether each item was reported, partially reported or not reported in the publication or was not applicable to the tool type. Horizontal lines separate the seven domains. Item-level justifications are given in Table S3.

Beyond illustration, we piloted the checklist on four published studies spanning the main tool classes (Section 2.4): the deep-learning prognostic model and the cluster-randomised decision-support trial just discussed [7,9], a large-language-model tool for identifying advance-care-planning documentation [38], and a co-designed mobile symptom-monitoring application from a low-resource setting [27]. Figure 3 shows the item-level results and Table S3 the justifications. Of 88 study-item pairs, 10 were rated reported, 32 partially reported, 31 not reported and 15 not applicable. Every item could be rated in at least one study, and no item was inapplicable across all four, which supports the applicability of the checklist across tool classes; not-applicable ratings clustered where they should, in the generative and preference items for non-generative tools and in the prognostic items for tools that make no prediction.

The pilot also shows where current reports fall silent. Three items were never adequately reported by any study to which they applied: end-of-life missingness and decedent data governance (item 13), the self-fulfilling prophecy safeguard (item 15) and answerability, regulatory class and drift monitoring (item 19). These are precisely the requirements that tend to live in governance files rather than in publications, which is an argument for asking authors to report them, not for waiving them. Two items drew on overlapping evidence when applied to prognostic models, the justified target (item 3) and access-abyss label bias (item 11); we retain both because they operate at different moments, item 3 as a design justification of what the target should be and item 11 as an empirical analysis of what the chosen label in fact encodes. The co-designed application, the only tool developed with sustained patient and family involvement, was also the only one rated reported for co-design, cultural safety and community integration, while the model-centred studies concentrated their reporting in the purpose and data domains; the checklist thus discriminates between tool cultures, not only between tools.

### 3.11. Novelties and Contributions

It is fair to ask what in PALLI-AI is genuinely new. Three concepts are, to our knowledge, original to this framework as operationalised requirements: the self-fulfilling prophecy safeguard as a reportable design item (item 15); the access-abyss label as a named, testable bias mechanism specific to a field defined by unmet need (item 11); and non-abandonment as a measurable design requirement, with time in human presence as its currency (item 6). A second group recontextualises palliative care scholarship for AI: total-suffering scope (item 4), dignity and personhood (item 5), family and bereavement as subjects and data holders (items 9 and 13), cultural and disclosure safety (item 10), pathway-initiation safeguards drawn from the Liverpool Care Pathway experience (item 18) and validation on goal-concordant care and quality of dying (item 20). A third group adapts established AI principles with a palliative sharpening: co-design (item 8) strengthens patient involvement from encouraged to required and adapts it to frailty; representativeness (item 12) narrows generic fairness to the trajectories this field serves; fabrication checks (item 16) bind a general concern of generative models to documented wishes; answerability and drift monitoring (item 19) specialise existing oversight duties with a hastened-death signal; and open science (item 22) is deliberately generic. The dual researcher–developer lens and the six named failure modes are, as organising devices, the framework’s principal methodological contribution.

## 4. Discussion

### 4.1. Principal Contribution

PALLI-AI translates the general requirements for trustworthy clinical AI into the specific context of palliative and end-of-life care and expresses each as both a reporting item and a design requirement. Its contribution is not an algorithmic method but a structured account of what a tool in serious illness must attend to, derived from the tensions of the setting rather than adapted from other specialities.

The framework is complementary to, not a replacement for, existing guidelines, and Table 2 makes the boundary explicit. TRIPOD+AI governs how a prediction model is reported, CONSORT-AI and SPIRIT-AI govern trials and their protocols, and DECIDE-AI governs early live evaluation [16,17,18,19,20]; a palliative prognostic study should still follow the relevant one. What those guidelines do not ask about is the scope of suffering, a target set by goals of care, non-abandonment, prognostic communication, the algorithmic surrogate and protocol rigidity, the requirements that become visible across serious illness and sharpen as the end-of-life approaches. PALLI-AI adds these and, through its dual lens, states them as design decisions a developer can act on before any study is written.

What distinguishes the proposal from a relabelling of generic guidance is set out in Section 3.11; its grounding now includes a structured and reproducible search (Section 2.1), an evidence-to-item matrix with certainty grades (Table S2) and a pilot application to published tools (Section 3.10). The framework remains method-agnostic, so it survives the shift from regression to foundation models.

### 4.2. Primary Function and Intended Use

PALLI-AI is, in order of present readiness, three things. It is first a reporting checklist: authors of studies of AI in palliative care can state, item by item, what their report addresses, and the pilot shows the exercise is feasible on published work. It is second a design guide: the developer lens states the same requirements as decisions that can be taken before any study exists. It is not yet an appraisal instrument, a scoring system or a governance standard: it carries no thresholds, weights or pass marks, and using it to rank or exclude tools would outrun its validation. After consensus refinement and reliability testing it could support structured appraisal, and its accountability domain may inform, but does not replace, regulatory classification [21,22].

### 4.3. Implementation Challenges

Using the checklist in practice will meet predictable obstacles. The first is burden: 22 items added to existing guidelines invite checklist fatigue, which is why each item is phrased as a single reportable sentence and why the pilot measured applicability. The second is incentive: reporting checklists change practice when journals and funders adopt them, so uptake will depend on endorsement as well as merit. The third is context: palliative services are under-resourced, their data infrastructure is thin, and several items, drift monitoring and decedent data governance among them, presuppose institutional capacity that a hospice may lack; the developer lens deliberately shifts part of that burden upstream to those who build the tools. The fourth is the box-ticking risk the framework itself names as protocol rigidity: a checklist completed mechanically can certify exactly the harm it was written to prevent. The remedy is judgement supported by structure rather than replaced by it, and periodic evaluation of whether the checklist changes what is designed and reported rather than merely what is claimed.

### 4.4. Adaptation to Low- and Middle-Income and Culturally Diverse Settings

Most unmet palliative need lies in low- and middle-income countries [4,6], yet most of the evidence behind this framework, like most AI in medicine, originates in high-income systems. Adaptation is therefore a first-order requirement, not an afterthought. Several items translate directly but change emphasis: representativeness (item 12) becomes representativeness of settings without electronic records; sustainability in under-resourced services (item 21) becomes the entry condition; equitable scale-up (item 22) becomes the primary outcome rather than the last item. The co-designed application in our pilot shows what adaptation looks like in practice: mobile-first delivery, community health workers and volunteers inside the workflow, and disclosure norms and spiritual framing configured locally rather than hard-coded [27]. Cultural adaptation is not confined to low-income settings: norms of prognostic disclosure and family decision-making vary within and between high-income societies, which is why cultural and disclosure safety (item 10) is a configurable requirement rather than a default. The planned consensus process reserves places for participants from low- and middle-income countries, and translation and local piloting of the checklist are part of the validation plan (Section 4.6).

### 4.5. Limitations

The proposal has limitations that we state plainly. First, the review beneath it, although now supported by a reproducible search and explicit criteria, remains narrative and purposive; no protocol was registered, and no formal quality-appraisal instrument was applied to individual sources, for which our A-to-C certainty grades are a partial substitute, not an equivalent. Second, the framework was developed by three authors from one national society, and no patients, family caregivers, bereaved people, developers, ethicists or regulators have yet taken part; this is the most important limitation of the present version and the reason the consensus stage precedes any claim to standardhood. Third, the pilot is small, confined to reporting rather than conduct, rated by the authors rather than by independent raters, and without reliability statistics. Fourth, the evidence for AI in palliative care is itself immature, dominated by feasibility studies and mortality prediction [8,10], so several items rest on level-C reasoning and analogy. Fifth, most cited work originates in high-income settings, a constraint examined in Section 4.4. Finally, a checklist can invite box-ticking, the failure that ended the Liverpool Care Pathway [43]; PALLI-AI is a support for judgement, not a substitute for it.

### 4.6. A Roadmap for Validation and Future Work

We commit to a concrete sequence rather than a gesture at future work. Step one is consensus: an international Delphi study conducted according to EQUATOR guidance for guideline development [23], with a panel of 30 to 45 participants drawn from eight groups, palliative care physicians and nurses, patients and family members including bereaved caregivers, methodologists, AI developers, ethicists and regulators, and with at least a third of the panel from low- and middle-income countries; two to three rounds; items rated on a nine-point scale under a priori rules, retention at seventy-five percent agreement or higher, revision between rounds with anonymised feedback, and unresolved items settled at a consensus meeting. Step two is reliability and usability: application of the refined checklist to at least twenty published and in-development tools by two independent raters per tool, reporting item-level weighted kappa, time to complete and rater-judged clarity, with items revised or merged where agreement fails. Step three is external validation and implementation: prospective use in new studies and across settings, including low- and middle-income services, measuring whether completeness of reporting improves against pre-checklist baselines and whether developers change design decisions, the outcome that matters most. The checklist will be revised on a stated cycle, every three years or upon a major technological shift, whichever comes first. The metrics of success are named in advance: inter-rater agreement, change in completeness of reporting, uptake by journals and developers, and user-rated utility.

## 5. Conclusions

AI is entering palliative and end-of-life care faster than the guidance needed to govern it. PALLI-AI answers with seven domains and 22 items. Each item is at once a reporting requirement for researchers and a design requirement for developers. Six named failure modes give the field a shared vocabulary for its characteristic errors. The proposal now rests on a structured and reproducible search, an evidence-to-item matrix with certainty grades, and a pilot application to four published tools. The pilot shows that the checklist can be applied to real studies. It also shows where current reports fall silent: data governance after death, safeguards against the self-fulfilling prognosis, and answerability. PALLI-AI is not a finished standard. It is an evidence-informed starting point for the Delphi consensus, reliability testing and external validation set out here. Its test will be practical: whether tools designed and reported with it serve people approaching the end of life, and their families, better.

## Figures and Tables

**Table 1:** The PALLI-AI checklist: twenty-two items across seven domains, each expressed as a reporting item (researcher lens) and a design requirement (developer lens).

No.	Checklist Item	Researcher Lens-Report in the Manuscript	Developer Lens-Build into the Tool
**P-Purpose set by goals of care**			
1	**Palliative problem and linked action**	State the palliative problem and the comfort or goal-of-care action the output triggers; a bare prognosis is not a purpose.	Tie every output to an actionable palliative response, not to a number.
2	**Point in the illness trajectory**	State where in the trajectory the tool acts, from early integration alongside disease-directed treatment to the last phase of life.	Design for that point in the trajectory; support early, concurrent palliative care, not only terminal care.
3	**Justified target, not a proxy**	Justify the target against goals of care; state that survival, cost or use are not the sole proxy for need.	Encode goal-concordance as the target; refuse cost or survival as a stand-in.
**A-Alignment with total suffering and dignity**			
4	**Total-suffering scope**	State which dimensions (physical, psychological, social, spiritual) are addressed and declare any single-dimension proxy.	Support the dimension you can address honestly; flag total-pain reduction as a limit.
5	**Dignity and personhood**	Report how the tool preserves dignity and avoids reducing the person to a score.	Design language and outputs that address a person, not a case.
6	**Non-abandonment**	Report the tool’s effect on time in human presence; lost contact is a harm, not a saving.	Route saved time back to the bedside; never let automation displace the clinician from the person and family.
7	**Direction and error asymmetry**	State whether the tool drives comfort and de-escalation or intervention and justify the cost of each error type.	Set thresholds so errors fail toward the patient’s goals, not toward escalation or withdrawal.
**L-Lived experience, family and bereavement**			
8	**Co-design with the seriously ill**	Report involvement of patients, families and clinicians, with methods adapted to frailty and attrition.	Co-design from the outset with methods a dying person can take part in.
9	**Family and bereavement as subjects**	Report whether family and caregiver outcomes and data, including after death, were addressed.	Treat the family as a unit of care and a data subject, into bereavement.
10	**Cultural and disclosure safety**	Report how disclosure norms, spiritual care and death practices were reflected.	Make disclosure and spiritual framing configurable, not hard-coded.
**L-Learning data and the equity of access**			
11	**Access-abyss label bias**	Analyse whether the outcome label encodes existing under-referral rather than true need.	Choose labels and cohorts that do not reproduce who currently gets access.
12	**Representativeness across trajectories**	Report age, frailty, multimorbidity, ethnicity, diagnosis and setting versus the target.	Curate data across trajectories and settings, not just cancer inpatients.
13	**End-of-life missingness and data governance**	Report missing-not-at-random data near death and governance of decedent and family data.	Handle end-of-life missingness explicitly; govern posthumous and family data lawfully.
**I-Interpretability and prognostic communication**			
14	**Communicable, honest prognosis**	Report how the output is rendered for a patient or family, who delivers it, and its calibration.	Output prognosis in a communicable, calibrated, uncertainty-honest form.
15	**Self-fulfilling prophecy safeguard**	State how the tool prevents its prediction from causing the outcome via premature withdrawal.	Break the feedback loop; do not let the prediction silently trigger withdrawal.
16	**Fabrication and preference fidelity**	For generative tools, report provenance, a hallucination check, and fidelity to documented wishes.	Ground outputs in the record; test for hallucination; never invent a preference.
**A-Accountability in serious illness**			
17	**No algorithmic surrogate**	State that inferred preferences do not displace documented wishes or the surrogate, and how capacity is handled.	Support, never replace, the surrogate; keep documented wishes authoritative.
18	**Pathway-initiation safeguards**	Report the authorisation, audit and override required before any output starts or withdraws an end-of-life pathway.	Require clinician authorisation and reversible, audited actions; never a mandatory protocol.
19	**Answerability, regulation and drift**	State who is answerable, the regulatory class, and monitoring for drift and hastened-death signal.	Assign a named owner; monitor safety and drift after deployment.
**I-Implementation and palliative outcomes**			
20	**Validation on palliative outcomes**	Report validation against goal-concordant care, quality of life and symptom burden across the illness course, place-of-death concordance and quality of dying, not accuracy alone.	Judge the tool on goal concordance and quality of life, not AUC.
21	**Interdisciplinary and community integration**	Report integration into the whole team and into home and hospice, and sustainability in low-resource services.	Fit the tool to the team and to home and hospice, not only the hospital.
22	**Equitable scale-up and open science**	Report reach across under-served groups, registration, code and model availability, and how AI was used.	Design for equitable, feasible scale-up; report transparently and reproducibly.

*AUC, area under the curve. Bold type marks the checklist item names; the shaded rows mark the seven domains.*

**Table 2:** What PALLI-AI adds beyond generic AI reporting guidelines, by domain.

Domain	What Generic AI Guidelines Already Cover	The Palliative-Specific Addition
**Purpose set by goals of care**	Intended use, task definition, target and predictors [16,17,18,19,20].	Output tied to a comfort or goal action; placement in the trajectory, from early integration to the last phase; target justified as goal-concordance, not survival or cost.
**Alignment with total suffering and dignity**	Intended use and, in trials, the comparator; no account of values.	Total-suffering scope; dignity; non-abandonment; error asymmetry set toward the patient’s goals.
**Lived experience, family and bereavement**	Patient involvement encouraged, not required.	Co-design designed for the frail and dying; family and bereavement as subjects and data holders; cultural and disclosure safety.
**Learning data and the equity of access**	Sample, predictors, missing data, subgroup fairness [16].	Access-abyss label bias; representativeness across trajectories and non-cancer settings; end-of-life missing-not-at-random; decedent and family data governance.
**Interpretability and prognostic communication**	Model transparency, calibration, explanation method.	Prognosis rendered for communication and who delivers it; self-fulfilling-prophecy safeguard; fidelity to documented wishes for generative tools.
**Accountability in serious illness**	Human oversight, risk analysis, regulatory status, monitoring.	No algorithmic surrogate; pathway-initiation authorisation and audit; monitoring for a hastened-death signal.
**Implementation and palliative outcomes**	Validation and performance; early live use in DECIDE-AI [19,20].	Validation on goal-concordant care, quality of life and of dying; interdisciplinary and home or hospice integration; scale-up judged by equity of access.

*Domain names are shortened; full titles appear in* Section 3.1 *. Bold type marks the domain names; the shaded header row follows the journal table style.*

**Table 3:** The six failure modes operationalised: working definition, observable indicators, example and counterexample, and a decision rule for applicability. Bracketed numbers are references. Bold type marks the failure-mode names.

Failure Mode	Working Definition	Observable Indicators	Example/Counterexample	Decision Rule
**Self-fulfilling prognosis**	A pessimistic prediction contributes to causing the death it forecasts: care is withdrawn and the death is read as confirmation.	Outcome includes deaths mediated by withdrawal decisions; no separation of physiology from decision-making; withdrawal rates rise after deployment.	Example: models predicting withdrawal of life-sustaining therapy, where centre culture can drive the decision [37]. Counterexample: an alert that triggers palliative consultation only, with treatment decisions expressly reserved to the team [9].	Applies when the predicted outcome can be produced by a decision the prediction influences. If so, item 15 is mandatory.
**Algorithmic surrogate**	A tool’s inference of what the patient would want displaces documented wishes or the human surrogate.	Output phrased as the patient’s preference; no precedence rule for documented wishes; intended use when capacity is lost.	Example: patient preference predictors proposed for incapacitated patients [40,42]. Counterexample: a language model that retrieves and summarises documented advance care planning with provenance [38].	Applies when any output represents or infers preferences. If so, items 16 and 17 are mandatory.
**Total-pain reduction fallacy**	Multidimensional suffering is collapsed into one measurable proxy, and the proxy is reported as the whole.	‘Need’ or ‘suffering’ operationalised as a single scale; spiritual and social dimensions absent from data and outcomes.	Example: benefit claimed on a physical symptom score alone [4,25]. Counterexample: a monitoring app addressing physical, emotional, spiritual and family dimensions explicitly [27].	Applies when the tool claims to detect or relieve suffering or need. If so, item 4 must declare the proxy.
**Access-abyss label**	A model trained on who currently receives care encodes under-referral of the under-served as ground truth.	Label is a service event (referral, enrolment) rather than a clinical state; cohort drawn from well-served settings; no under-served subgroup analysis.	Example: a cost proxy that under-selected Black patients until the target was corrected [30]. Counterexample: a mortality window justified explicitly as a screening proxy and audited by chart review [7].	Applies when the label may encode access rather than need. If so, item 11 analysis is mandatory.
**Abandonment by automation**	Efficiency gains reduce the human presence that is itself part of the treatment.	Time savings claimed without stating where the saved time goes; automation of patient-facing communication at sensitive moments.	Example: automated communication displacing human contact, warned against across the field [14,26]. Counterexample: remote monitoring experienced by patients as increased presence [27].	Applies when the tool automates contact, communication or triage. If so, item 6 is mandatory.
**Protocol rigidity**	A tool hardens into a mandatory pathway that overrides individual judgement.	Default actions without override; performance targets tied to pathway initiation; alerts that enrol patients automatically.	Example: the Liverpool Care Pathway, withdrawn after harms from protocolised application [43]. Counterexample: initiation requiring explicit clinician authorisation with a reversible, audited trail [9].	Applies when any output can start, escalate or withdraw a care pathway. If so, item 18 is mandatory.

## Data Availability

The data supporting this article (the full search strategy and record counts, the evidence-to-item matrix, and the item-level pilot ratings) are available in the Supplementary Materials (Tables S1–S3).

## Supplementary Materials

The following supporting information can be downloaded at: https://www.mdpi.com/article/10.3390/healthcare14182998/s1, Table S1: Full search strategy, PubMed query translations and record counts; Table S2: Evidence-to-item matrix linking each item to its tension, failure mode, sources and certainty grade; Table S3: Item-level pilot ratings with justifications.
