# Supplementary material for: The PALLI-AI Framework: An Evidence-Informed Checklist for Designing and Reporting Trustworthy Artificial Intelligence Tools in Palliative and End-of-Life Care
Source: Healthcare (Basel). 2026 Sep 14;14(18):2998. doi: 10.3390/healthcare14182998 (PMC13606585; doi:10.3390/healthcare14182998)
Supplement: Supplementary file 1 [file healthcare-14-02998-s001.zip › Supplementary_Materials_S1-S3_proofstage (1).pdf]

Supplementary Materials

The PALLI-AI Framework: An Evidence-Informed Checklist for Designing and Reporting Trustworthy Artificial Intelligence Tools in Palliative and End-of-Life Care

Mora-Delgado J., Rivas Jiménez V., Lojo-Cruz C.

Table S1. Full search strategy, PubMed query translations and record counts

Search executed in PubMed (National Library of Medicine) on **25 August 2026**, from database inception, with no language restriction and no publication-type filter. Counts are the exact totals returned by PubMed's E-utilities on the day of search; PubMed totals drift as records are added. PubMed's phrase normalisation collapses "end of life"[Title/Abstract] and "end-of-life"[Title/Abstract] to the same indexed term; this does not change the counts.

Block	Query (verbatim)	Records
Q1. Palliative and end-of-life care	("palliative care"[MeSH Terms] OR "terminal care"[MeSH Terms] OR "hospice care"[MeSH Terms] OR "hospices"[MeSH Terms] OR palliative[Title/Abstract] OR "end of life"[Title/Abstract] OR "end-of-life"[Title/Abstract] OR hospice[Title/Abstract] OR "terminally ill"[Title/Abstract])	186,341
Q2. Artificial intelligence	("artificial intelligence"[MeSH Terms] OR "machine learning"[MeSH Terms] OR "natural language processing"[MeSH Terms] OR "artificial intelligence"[Title/Abstract] OR "machine learning"[Title/Abstract] OR "deep learning"[Title/Abstract] OR "large language model"[Title/Abstract] OR "large language models"[Title/Abstract] OR "natural language processing"[Title/Abstract] OR chatbot[Title/Abstract] OR chatbots[Title/Abstract])	522,905
Q3. Core corpus (Q1 AND Q2)	Q1 AND Q2	872
Q3b. Core corpus, 2015–present	Q3 AND ("2015/01/01"[Date - Publication] : "3000"[Date - Publication])	829
Q4. Prognostic communication	("prognosis"[MeSH Terms] OR prognostic[Title/Abstract]) AND	19

Block	Query (verbatim)	Records
	(“communication”[MeSH Terms] OR communication[Title/Abstract]) AND (palliative[Title/Abstract] OR “end of life”[Title/Abstract] OR “serious illness”[Title/Abstract]) AND (“artificial intelligence”[Title/Abstract] OR “machine learning”[Title/Abstract] OR “large language model”[Title/Abstract])	
Q5. Surrogate decision-making and preference prediction	(“advance care planning”[MeSH Terms] OR “surrogate”[Title/Abstract] OR “advance care planning”[Title/Abstract] OR “goals of care”[Title/Abstract]) AND (“artificial intelligence”[Title/Abstract] OR “machine learning”[Title/Abstract] OR “large language model”[Title/Abstract] OR “patient preference predictor”[Title/Abstract])	1,796
Q6. Algorithmic bias and fairness in health care	(“algorithmic bias”[Title/Abstract] OR “algorithmic fairness”[Title/Abstract] OR (“bias”[Title/Abstract] AND algorithm[Title/Abstract])) AND (“healthcare”[Title/Abstract] OR “health care”[Title/Abstract] OR clinical[Title/Abstract])	2,799
Q7. Explainable artificial intelligence in medicine	(“explainable artificial intelligence”[Title/Abstract] OR “explainable AI”[Title/Abstract] OR “XAI”[Title/Abstract] OR interpretability[Title/Abstract]) AND (clinical[Title/Abstract] OR medicine[Title/Abstract] OR health[Title/Abstract]) AND (“machine learning”[Title/Abstract] OR “artificial intelligence”[Title/Abstract])	8,368
Q8. AI reporting guidelines (known-item block)	(“TRIPOD”[Title/Abstract] OR “CONSORT-AI”[Title/Abstract] OR “SPIRIT-AI”[Title/Abstract] OR “DECIDE-AI”[Title/Abstract]) AND (guideline[Title/Abstract] OR statement[Title/Abstract] OR checklist[Title/Abstract] OR extension[Title/Abstract])	476

Screening and selection. Records were screened on title and abstract against the eligibility criteria stated in Section 2.1 of the manuscript. Within eligible records, selection was purposive, as befits a narrative review: sources were chosen for their capacity to define or illustrate a palliative-specific requirement. Reference lists of key reviews were hand-searched. Screening and selection were performed by one author (J.M.-D.) and independently checked by the other two; disagreements were

resolved by discussion until consensus. The final evidence base comprises 43 peer-reviewed publications and 4 institutional documents (47 references).

Table S2. Evidence-to-item matrix linking each item to its tension, failure mode, sources and certainty grade

Each of the 22 checklist items linked to the palliative-specific tension it answers, the failure mode it guards against (where one applies), its key supporting sources (reference numbers as in the manuscript) and the certainty grade of the underlying evidence. Grades: **A** = direct comparative evidence (randomised or controlled studies bearing on the requirement); **B** = direct non-comparative evidence (observational, qualitative, feasibility or review studies in palliative populations); **C** = conceptual evidence (normative analyses, governance documents, or findings from adjacent fields applied by argument).

#	Item	Domain	Palliative-specific tension	Failure mode guarded	Key sources	Certainty
1	Palliative problem and linked action	P	The purpose is relief, not prediction; a bare prognosis is not a purpose	Drift toward bare prognostication	[1,7,9]	A
2	Point in the illness trajectory	P	Care begins at diagnosis and spans trajectories, not only the last days	—	[1,2,3]	A
3	Justified target, not a proxy	P	True need vs. measurable proxies (survival, cost, use)	Access-abyss label	[4,7,30]	B
4	Total-suffering scope	A	Suffering is physical, psychological, social and spiritual at	Total-pain reduction fallacy	[1,4,25]	B

#	Item	Domain	Palliative-specific tension	Failure mode guarded	Key sources	Certainty
			once			
5	Dignity and personhood	A	The person is not reducible to a score	Abandonment by automation	[1,26]	C
6	Non-abandonment	A	Human presence is itself part of the treatment	Abandonment by automation	[14,26]	C
7	Direction and error asymmetry	A	Errors toward escalation and toward withdrawal carry different harms	Self-fulfilling prognosis	[7,9]	B
8	Co-design with the seriously ill	L (lived exp.)	Involvement must be designed for people who can give only a little	—	[27,28,29]	B
9	Family and bereavement as subjects	L (lived exp.)	The family is a unit of care and a data subject, into bereavement	—	[1,27,28]	B
10	Cultural and disclosure safety	L (lived exp.)	Disclosure norms and death practices vary across cultures	—	[27,34]	B
11	Access-abyss label bias	L (data)	The label records who	Access-abyss label	[4,5,30]	B

#	Item	Domain	Palliative-specific tension	Failure mode guarded	Key sources	Certainty
			reached the service, not who needed it			
12	Representativeness across trajectories	L (data)	The palliative population is old, frail, multimorbid, often non-cancer	Access-abyss label	[12,31]	B
13	End-of-life missingness and data governance	L (data)	The sickest stop generating data; decedent and family data need governance	—	[22,34]	C
14	Communicable, honest prognosis	I (interp.)	A prognosis is communicated to a person as part of care	Self-fulfilling prognosis	[7,35,36]	B
15	Self-fulfilling prophecy safeguard	I (interp.)	A prediction can cause the outcome it forecasts	Self-fulfilling prognosis	[37]	B
16	Fabrication and preference fidelity	I (interp.)	Generative tools can invent; wishes must be grounded in the record	Algorithmic surrogate	[38,39]	B
17	No algorithmic surrogate	A (account.)	Satisfying an inferred preference is not	Algorithmic surrogate	[40,41,42]	C

#	Item	Domain	Palliative-specific tension	Failure mode guarded	Key sources	Certainty
			respecting autonomy			
18	Pathway-initiation safeguards	A (account.)	A protocol that overrides judgement harms; the Liverpool Care Pathway lesson	Protocol rigidity	[43]	B
19	Answerability, regulation and drift	A (account.)	Oversight duties become acute where a hastened-death signal is possible	Protocol rigidity	[21,22,44,45]	C
20	Validation on palliative outcomes	I (implem.)	The outcomes that matter are goal-concordance, quality of life and of dying	—	[2,9,47]	A
21	Interdisciplinary and community integration	I (implem.)	Care is delivered by a team across hospital, home and hospice	—	[10,13,25]	B
22	Equitable scale-up and open science	I (implem.)	Most unmet need lies where resources are least	Access-abyss label	[4,6]	B

Distribution: 3 items at level A (items 1, 2, 20), 14 at level B, 5 at level C (items 5, 6, 13, 17, 19).

Table S3. Item-level pilot ratings with justifications

Four published studies, one per tool class: **S1** deep-learning prognostic model (Avati et al. [7]); **S2** cluster-randomised trial of an AI decision-support tool (Wilson et al. [9]); **S3** large-language-model documentation tool (Agaronnik et al. [38]); **S4** co-designed mobile symptom-monitoring application (Carey et al. [27]). Ratings: **R** = reported; **P** = partially reported; **N** = not reported; **n/a** = not applicable to the tool type. Ratings judge completeness of reporting in the publication, not quality of the work.

Item	S1 (prognostic model)	S2 (decision-support trial)	S3 (LLM tool)	S4 (co-designed app)
1. Palliative problem and linked action	**R** — under-referral problem stated; prediction triggers proactive palliative outreach	**R** — need prediction and triggered consultation stated	**P** — tracking purpose stated; no patient-level action linked	**P** — unmet symptom burden stated; action pathway loosely specified
2. Point in the illness trajectory	**R** — explicit 3–12-month window at admission	**P** — hospitalised setting stated; trajectory position not stated	**P** — advanced cancer population; the tool's role implicit	**P** — hospice/day-care patients; stage not explicit
3. Justified target, not a proxy	**R** — mortality framed as proxy; appropriateness audited by chart review	**N** — derivation of the “need” target not reported	**R** — target is ACP documentation itself, goal-of-care aligned	**n/a** — no prediction target; symptoms monitored directly
4. Total-suffering scope	**P** — proxy declared; suffering dimensions not stated	**N** — dimensions not reported	**P** — four ACP domains declared; suffering not framed	**R** — physical, emotional, spiritual, family dimensions explicit

Item	S1 (prognostic model)	S2 (decision-support trial)	S3 (LLM tool)	S4 (co-designed app)
5. Dignity and personhood	**N**	**N**	**N**	**P** — holistic, culturally personalised framing; dignity not explicit
6. Non-abandonment	**N** — effect on human presence not reported	**P** — consultation rate increased; contact time unmeasured	**n/a** — retrospective documentation tool	**P** — qualitative reports of presence ("a doctor beside me")
7. Direction and error asymmetry	**P** — precision-first thresholds; error costs not fully justified	**P** — drives consultation; asymmetry not reported	**P** — false-positive relevance discussed; costs unjustified	**P** — comfort direction clear; error costs not discussed
8. Co-design with the seriously ill	**P** — clinician review only; no patient/family involvement	**N**	**N**	**R** — two-phase co-design with patients, carers, workers
9. Family and bereavement as subjects	**N**	**N**	**N**	**P** — carers involved; no bereavement data
10. Cultural and disclosure safety	**N**	**N**	**N**	**R** — three religions, Amharic, "hidden patient", disclosure norms
11. Access-	**P** — counters	**N**	**N** —	**n/a** — no

Item	S1 (prognostic model)	S2 (decision-support trial)	S3 (LLM tool)	S4 (co-designed app)
abyss label bias	referral bias; no formal label-bias analysis		documentation label may encode practice gaps; not analysed	trained outcome label
12. Representativeness across trajectories	**P** — all-comers cohort; ethnicity/frailty vs. target unexamined	**P** — sites and cohort given; demographics not detailed	**P** — 60 patients, 7 clinics; demographics not detailed	**P** — ages, diagnoses, settings given; single hospice
13. End-of-life missingness and governance	**N**	**N**	**P** — secure infrastructure stated; MNAR/decedent governance absent	**P** — ethics and consent each stage; no decedent governance
14. Communicable, honest prognosis	**P** — calibration reported; patient-facing communication not described	**N**	**n/a** — no prognostic output to patients or families	**n/a** — no prognostic output
15. Self-fulfilling prophecy safeguard	**N**	**N**	**n/a** — no outcome prediction	**n/a** — no prediction
16. Fabrication and preference fidelity	**n/a** — discriminative model	**n/a** — discriminative tool	**R** — hallucination index, provenance checking reported	**n/a** — curated content, not generative
17. No algorithmic surrogate	**n/a** — predicts mortality, not preferences	**n/a** — predicts service need	**P** — anchored to documented wishes; displacement/capacity unstated	**n/a** — no preference inference
18. Pathway-initiation	**P** — clinician in the loop;	**P** — recommendat	**n/a** — no care-	**n/a** — prototype

Item	S1 (prognostic model)	S2 (decision-support trial)	S3 (LLM tool)	S4 (co-designed app)
safeguards	no audit/override detail	ion implies clinician decision; no detail	pathway output	initiates no pathway
19. Answerability, regulation and drift	**P** — drift and retraining discussed; answerability absent	**N**	**N**	**N** — risk testing deferred
20. Validation on palliative outcomes	**N** — accuracy metrics only	**P** — consultations, readmissions, length of stay; no QoL or goal-concordance	**N** — accuracy vs. gold standard only	**N** — user satisfaction only; effectiveness pending
21. Interdisciplinary and community integration	**P** — workflow integration with palliative team; no community reach	**P** — "integrated into practice"; no detail	**N** — integration deferred	**R** — hospice, home, community volunteers, sustainability
22. Equitable scale-up and open science	**N** — no registration, code or equity analysis	**N**	**N**	**P** — strong equity framing; no registration or open materials

Summary. Of 88 study-item pairs: 10 reported, 32 partially reported, 31 not reported, 15 not applicable. Per study (R/P/N/n-a): S1 3/9/8/2; S2 1/7/12/2; S3 2/7/9/4; S4 4/9/2/7. Items 13, 15 and 19 were never adequately reported by any study to which they applied. Items 3 and 11 drew on overlapping evidence for prognostic models; their distinct scopes (design justification vs. empirical label analysis) are stated in Section 3.10 of the manuscript.
